# Establishment and validation of an orthotopic brain metastasis tumor model in C57BL/6 mice

**DOI:** 10.7717/peerj.20913

**Published:** 2026-03-26

**Authors:** Jiong Liu, Tonghao Lin, Juan Wang, Jiayun Hao, Wencheng Wu, Zhixin Zheng, Haibin Ou, Yu Liu

**Affiliations:** Department of Esophagus, Mediastinum and Lymphatic Oncology, Zhongnan Hospital of Wuhan University, Wuhan, Hubei Province, China

**Keywords:** Brain tumor, Animal model, C57BL, Intracranial tumor formation, Standardized procedure, Neurosurgical techniques, Xenograft, Brain metastases, LLC

## Abstract

As a key experimental method to study the pathogenesis of brain tumors, the establishment of standardized operation process for intracranial orthotopic tumor modeling is of great significance. At present, although this technology has become the main method for brain tumor research, it has been reported in the literature that there is significant heterogeneity in the injection sites, and there is a lack of systematic verification, which leads to the repeatability of the experiment being questioned. In view of this technical bottleneck, this study used female C57BL mice as a model to establish a standardized intracranial tumor formation operation system. By systematically analyzing the two anatomical markers of bregma and lambda in mice, we not only clarified the quantitative relationship between body weight and bregma-lambda distance in C57BL mice, but also defined the normal range of bregma-lambda distance. More importantly, we found and verified a new standardized injection site. Based on this finding, this study finally constructed a complete set of intracranial tumor formation technology scheme including positioning method, operation specification and quality control.

## Introduction

Brain tumors can be classified into two major categories: primary brain tumors and secondary brain tumors. Primary brain tumors refer to neoplasms originating in the central nervous system. In adult populations, the annual incidence of primary malignant brain tumors is approximately 7 per 100,000 individuals, with glioblastoma multiforme (GBM) accounting for about 50% of cases. GBM is a highly aggressive malignant tumor characterized by a median patient survival of merely 12–18 months. Other common types of primary brain tumors include primary central nervous system lymphoma (PCNSL), malignant meningioma, and ependymoma, among others (Wang, 2023). In pediatric patients, medulloblastoma and ependymoma represent the most prevalent types of primary brain tumors (Schakelaar et al., 2023). In contrast, the incidence of secondary brain tumors (also known as brain metastases, BM) is 10 times higher than that of primary brain tumors, originating from intracranial spread of solid tumors in other organs. Epidemiological data indicate that 10%–40% of cancer patients will develop brain metastases during the course of their disease (Lamba, Wen & Aizer, 2021). Approximately 50% of patients succumb within 3–27 months following brain metastasis diagnosis (Ferguson et al., 2018). The predominant primary tumors responsible for brain metastases are non-small cell lung cancer (NSCLC), breast cancer, and melanoma (Achrol et al., 2019). Median survival periods post-diagnosis are 7–12 months, 14–16 months, and 7–10 months for these tumor types, respectively (Ferguson et al., 2018; Sperduto et al., 2020).

Currently, radiotherapy remains one of the primary treatment modalities for both primary and secondary brain tumors. With advancements in medical science, the combined application of immunotherapy and targeted therapy has brought new hope to patients. However, the optimal combination strategies and sequencing of these treatment regimens still require further validation of their efficacy and safety through large-scale clinical trials. Given the current limitations and suboptimal outcomes of brain tumor therapies, in-depth research into the pathogenesis of brain tumors and exploration of novel treatment approaches are of paramount importance. Research on brain tumor mechanisms and treatment efficacy relies on reliable tumor modeling methods. Currently established approaches include: stereotactic intracranial orthotopic injection, intracardiac injection, and carotid artery injection and freehand intracranial injection (Daviaud et al., 2025; Liu et al., 2019; Qi et al., 2024; Zhang, Lowery & Yu, 2017). Stereotactic intracranial injection offers moderate technical difficulty with consistent tumor formation, but intracardiac injection may cause extracranial metastases and experimental animal mortality (Liu et al., 2019; Lowery & Yu, 2017). Carotid artery injection can lead to contralateral brain metastasis or facial tumors (Zhang, Lowery & Yu, 2017), with potential vascular occlusion and stroke (Lim et al., 2022). Freehand intracranial injection is a relatively recent technique with limited adoption, and it still lacks standardized guidance for targeting specific brain regions (*e.g.*, striatum), resulting in restricted positioning accuracy (Qi et al., 2024). Given the limitations of various tumor modeling approaches, stereotactic intracranial orthotopic implantation has become the most widely used method in current research. However, a PubMed search reveals that detailed protocols for this technique remain scarce in the literature, and the reproducibility of reported intracranial tumor locations continues to be questionable. Moreover, the skull anatomy varies significantly across different mouse strains, making it challenging to standardize intracranial injection sites. Even when targeting the same anatomical location within the same strain, substantial variations in injection coordinates persist across published studies. This lack of consistency poses significant challenges for neuroscientists conducting related research. This situation urgently calls for standardized protocols to guide intracranial tumor modeling procedures, thereby facilitating advancements in brain tumor research.

To address these challenges, our research team has developed a standardized intracranial tumor modeling protocol using C57BL/6 mice as a model system. This established model enables researchers to: (1) Recapitulate the growth and invasion patterns of primary or metastatic human brain tumors, facilitating in-depth investigation of tumor biology and molecular mechanisms (Fels-Palesandro et al., 2025). (2) Evaluate the efficacy and safety of novel antitumor agents for clinical translation (Cobb et al., 2022). (3) Investigate tumor-brain microenvironment interactions, including immune cell infiltration, angiogenesis, and neuroinflammatory processes (Farhadi et al., 2005; Kodack et al., 2017). (4) Develop and optimize innovative therapeutic strategies (*e.g.*, immunotherapy, targeted therapy, and combination regimens) (Kodack et al., 2012; Ni et al., 2016). (5) Elucidate mechanisms of tumor metastasis to the brain and the microenvironmental requirements for intracranial survival and proliferation.

## Materials & Methods

### Preparation

#### Ethical approval

Our experimental procedures were approved by the Laboratory Animal Welfare and Ethics Committee of Zhongnan Hospital, Wuhan University (Approval No. ZN2023219).

#### Biological preparation

Female C57BL/6 mice (≥8 weeks old, 20–30 g) were obtained from Ente Biotechnology Co., Ltd. (Hubei, China) and housed under specific pathogen-free (SPF) conditions (22 ± 1 °C, 40%–60% humidity, 12 h light/dark cycle) with ad libitum access to autoclaved feed and sterile water. A maximum of five mice were housed per individually ventilated cage (IVC), provided with sterilized hardwood chew blocks and corncob bedding changed twice weekly. LLC cells (Wuhan Zishan Biotechnology Co., Ltd.) and luciferase-stably transfected LLC cells (generated in-house, Supplemental File S5) were cultured in DMEM (10% FBS, 1% P/S) at 37 °C/5% CO_2_, with all cells used within 10 passages. Cells were harvested for experimentation when reaching 70–80% confluence.

#### Instrument preparation

**Figure 1:**
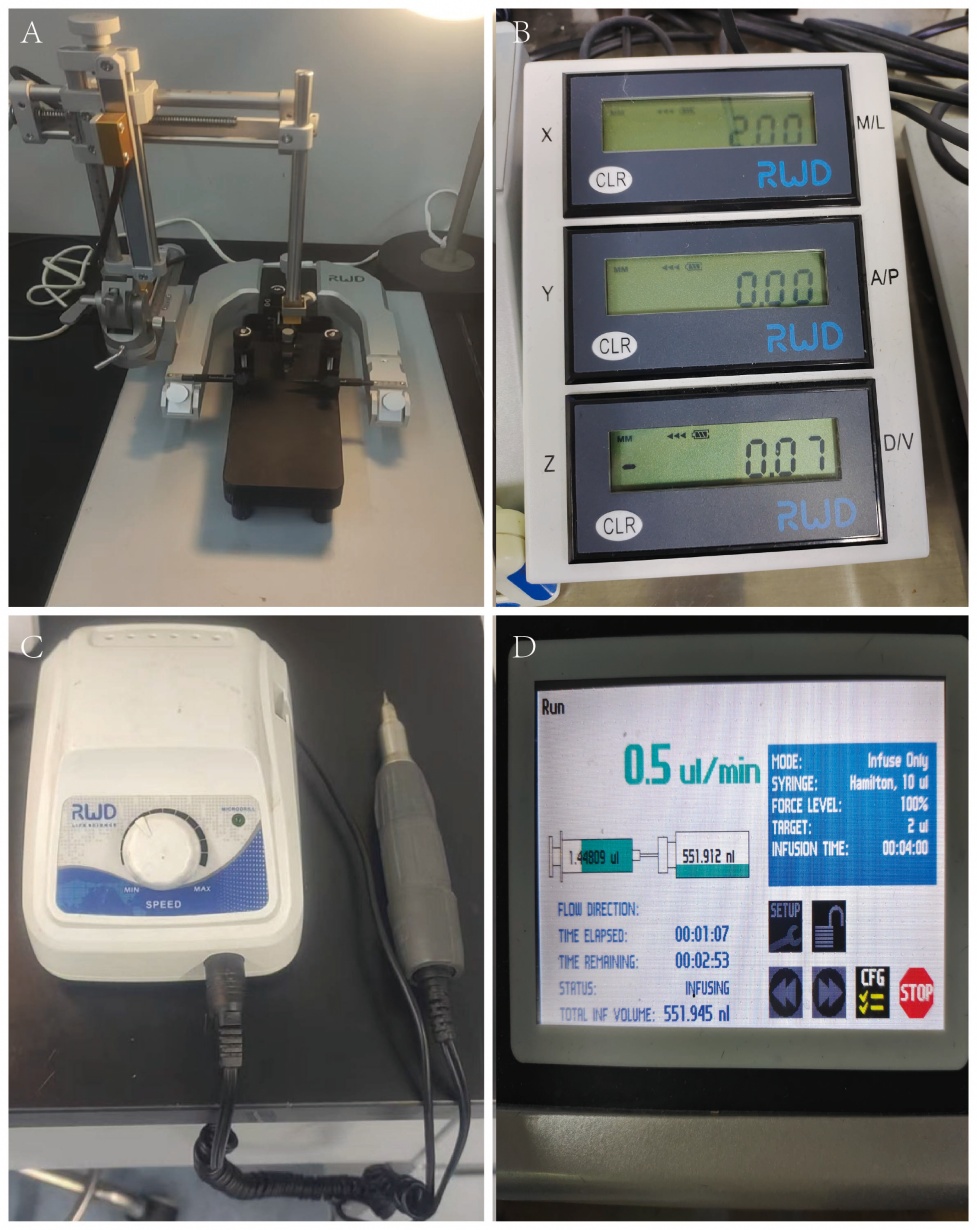
Components of a stereotaxic instrument for mouse intracranial injection. (A) Stereotaxic head holder for mice. (B) Matched positioning sensor with real-time XYZ-axis position display. (C) Matched cranial drill and drill bit set. (D) Matched microprocessor controlled microsyringe pump.

Standard stereotaxic instrument set with matching micro hand-held cranial drill (RWD Life Science, China) (Fig. 1); mouse surgical kit (including forceps, scissors, sterile suture needles with thread, needle holders, ophthalmic scissors, ophthalmic forceps, hemostats); 10 µL Hamilton microsyringe; Sterile marker pen; mouse-specific electric hair clippers; multifunctional vortex mixer; temperature-controlled heating pad; sterile cotton swabs; sterile surgical drapes; one mL and 20 ml syringes; disposable intravenous infusion needle; constant-temperature cryostat microtome; small animal *in vivo* imaging system.

#### Chemical preparation

Coomassie brilliant blue staining solution, 1% sodium pentobarbital (prepare fresh before use), 75% alcohol, phosphate-buffered saline (PBS), DMEM medium, fetal bovine serum, penicillin-streptomycin dual antibiotics, trypsin, erythromycin ointment, depilatory cream, perfusion saline solution (20 U/ml heparin saline), 4% paraformaldehyde fixative, 30% sucrose solution, OCT embedding agent, D-luciferin potassium salt.

#### Site and personnel preparation

Operate in SPF environment, prepare at least two personnel (master operator and assistant).

#### Operational preparations

Sterilize surgical instruments by autoclaving or glass bead dry-heat sterilization. Prepare cell suspensions at 2 ×10^5^/ µL and maintain on ice during transport. For Hamilton syringes, disassemble components and immerse fully in 75% ethanol for 5 min, followed by PBS soaking for 5 min. Air-dry before use.

### Qualified injection site localization and calibration methods

#### Mouse anesthesia

Anesthetize mice by intraperitoneal injection of 1% pentobarbital sodium (0.1 mL/20 g body weight; 50 mg/kg). Assess anesthesia 5–10 min post-injection *via* hind paw pinch with forceps. If reflexes persist, supplement with 0.03 mL/20 g (15 mg/kg) and re-evaluate after 5 min. Repeat if necessary.

#### Maintenance of body temperature

Place anesthetized mice on a thermostatic heating pad to maintain body temperature.

#### Mouse skin preparation

**Figure 2:**
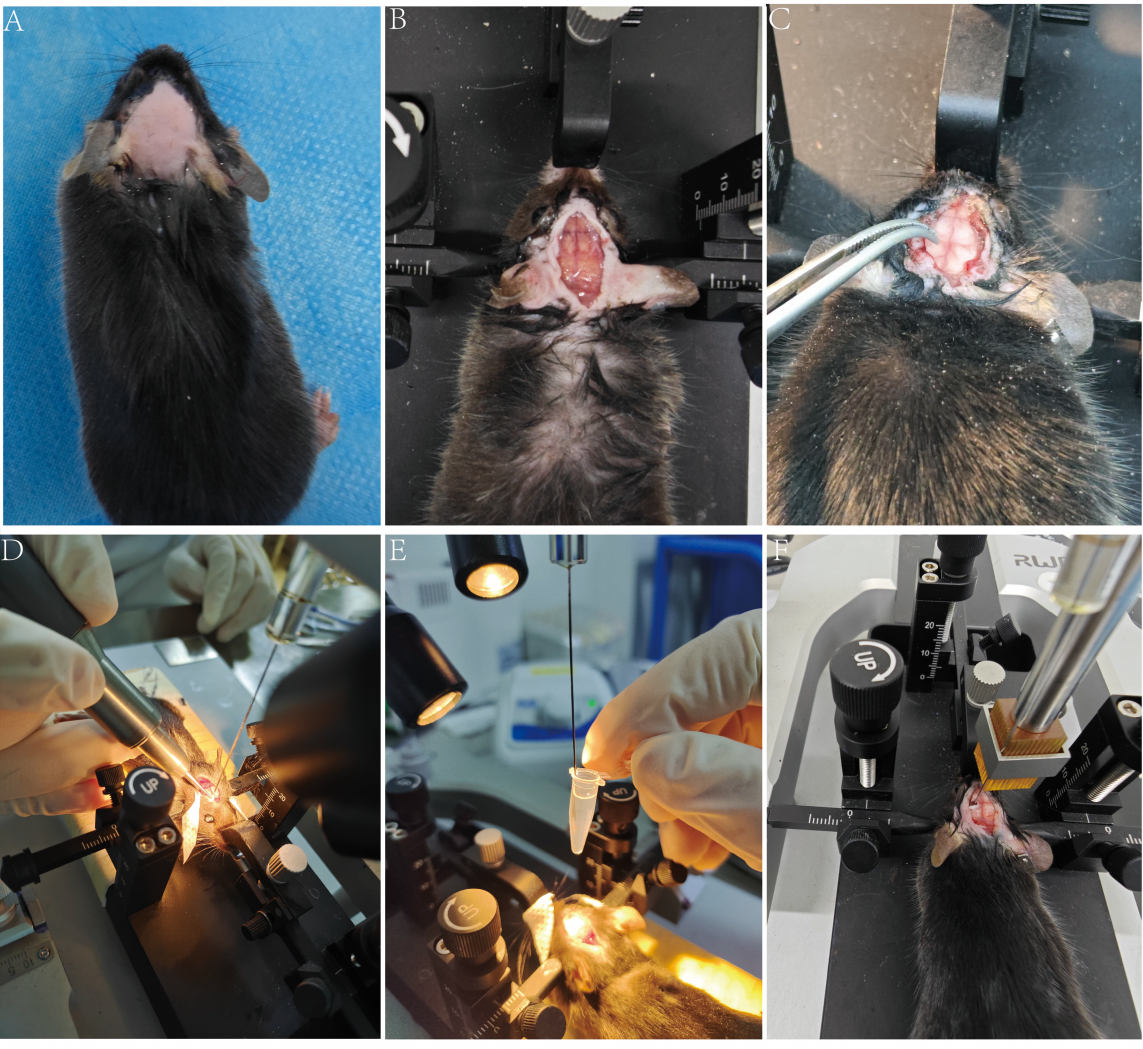
Schematic diagram of the general intracranial injection procedure. (A) Skin preparation. (B) Head fixation and scalp incision. (C) Identification of bregma and lambda. (D) Locate the drilling site and commence drilling. (E) Extract the cell suspension. (F) Inject the cell suspension.

Remove fur from the frontal to posterior neck region using rodent clippers. Apply depilatory cream evenly with a cotton swab, then gently wipe off after 30 s (Fig. 2A).

#### Head fixation

Secure the mouse’s upper incisors onto the bite bar. Adjust the nose clamp to gently press the nasal tip. Position the ear bars into the concave areas anterior to both ear canals. Horizontally align the ear bars so that the midpoint between the ears lies along the extension line of the nose clamp. Vertically adjust the ear bars and nose clamp to position the skull approximately parallel to the horizontal plane. Verify stability by gently pressing the head and lifting the tail to rotate the mouse. Fix repeatedly if instability is detected.

#### Scalp disinfection

Disinfect the mouse scalp three times using povidone-iodine or 75% alcohol.

#### Scalp incision

Make a 1.5 cm midline incision along the skull from the neck to the interocular midpoint (Fig. 2B).

#### Periosteum removal

Dissect the scalp with two sterile cotton swabs to expose the skull. Gently abrade the skull surface with the swabs to remove the periosteum.

#### Hamilton microsyringe installation

Mount a 10 µL Hamilton microsyringe onto the stereotaxic apparatus.

#### Bregma-lambda alignment and skull leveling

Position the micropipette tip at the bregma and zero the stereotaxic device. Then, move the tip to the lambda and adjust the nose bar height based on the *Z*-axis reading. Repeat this process until the *Z*-axis coordinates of the bregma and lambda differ by less than 0.03 mm. Next, move the tip to a point 2.00 mm lateral to the midpoint between the bregma and lambda, record the *Z*-axis coordinate, and adjust the left and right ear bars until the *Z*-axis readings on both sides differ by less than 0.03 mm. At this stage, the mouse skull plane is considered level. Note: Each half-turn of the nose bar or ear bars changes the *Z*-axis coordinate by approximately 0.07 mm.

#### Target site marking

Position the microsyringe tip at the predetermined target coordinates, then lift it slightly to mark the target on the skull with a sterile surgical pen.

#### Drilling

Use a skull drill to perform shallow drilling at the marked site (avoid complete penetration to prevent brain tissue damage), then puncture the remaining bone layer with a sterile syringe needle. If bleeding occurs, immediately absorb it with a sterile cotton swab (Fig. 2D).

#### Dye loading

Aspirate 1 µL coomassie brilliant blue dye using a Hamilton microsyringe.

#### Intracranial injection of coomassie brilliant blue

Position the Hamilton microsyringe at the puncture point. Lower the needle to the target depth (generally 3.0 mm below the skull surface). Move the syringe up and down 0.5 mm 3–5 times along the *Y*-axis direction to remove potential tissue obstruction. Re-position precisely at 3.0 mm depth. Inject dye slowly and steadily over 2 min (0.5 µL/min flow rate). Upon completion of injection, maintain a 1-minute dwell period to minimize reflux along the needle track.

#### Withdraw the Hamilton microliter syringe

Slowly retract the Hamilton micropipette and pause for 30 s at depths of 2 mm, 1 mm, and 0.5 mm respectively, to prevent liquid overflow.

#### Cardiac perfusion and brain extraction

Refer to (Article S1).

#### Brain tissue cryosectioning, measurement, and imaging

Perform serial cryosectioning of mouse brain (Article S1). Use a calibrated ruler to measure the X- and *Y*-axis coordinates of dye spread in the horizontal plane. Capture high-resolution images of injection sites (Figs. S1A–S1B).

#### Injection site evaluation and calibration

**Figure 3:**
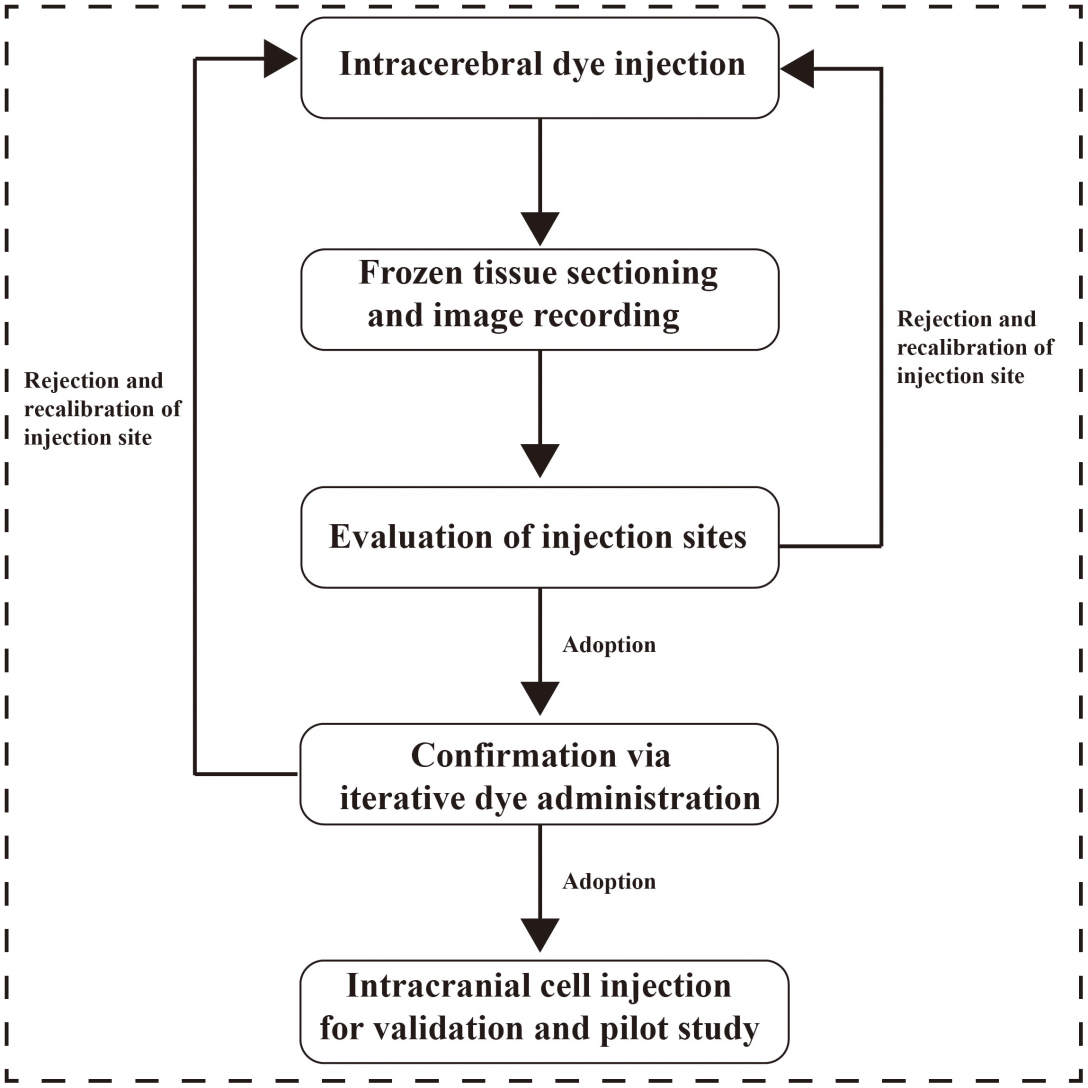
Injection site quality control workflow.

**Figure 4:**
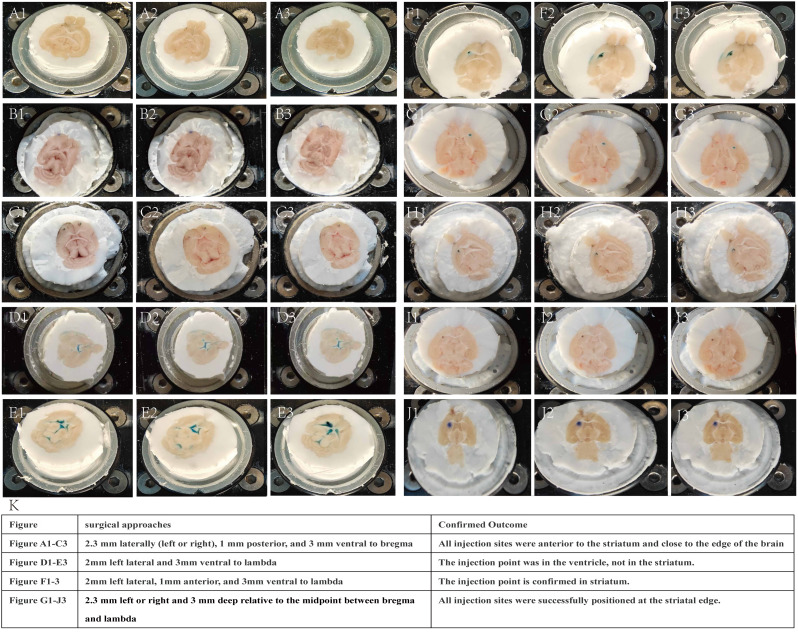
Stereotaxic dye mapping for intracranial target localization. (A, B) and (C) Injection site −2.3 mm laterally (left or right), one mm posterior, and three mm ventral to bregma. All injection sites confirmed by frozen sections were anterior to the striatum and close to the edge of the brain. In the cases of (A and B), the dye was faintly visible at the edges of the brain tissue. (D & E) Injection site −2 mm left lateral and three mm ventral to lambda. The injection point confirmed by frozen sections was in the ventricle, not in the striatum. (F) Injection site −2 mm left lateral, one mm anterior, and three mm ventral to lambda. The injection point is confirmed in striatum. (G, H, I) and (J) Injection site −2.3 mm lateral to bregma, at the bregma-lambda midpoint, and three mm in depth. The injection sites in these four mice (G, H, I, and J) showed remarkable consistency, all located near the border of the striatum. (K) Tabulated summary of stereotaxic coordinates and histologically verified injection sites using frozen sections.

Assess dye injection coordinates (Figs. 3 and 4). If the injection site is acceptable, proceed to the next step. If suboptimal, adjust stereotaxic coordinates based on the deviations (X, Y, Z) from the target, and repeat with steps 1–17 for verification (Fig. 3).

#### Validation of calibrated injection sites

Randomly select multiple mice to verify the injection site identified in Step 17. Upon successful validation, proceed to the pilot intracranial tumor formation experiment; otherwise, repeat Steps 1–18 (Fig. 3).

### Operating procedure for intracranial tumor modeling

#### Repeat steps 2.1 to 2.11

#### Cell suspension aspiration

Vortex cell suspension (10 s, 2,000 rpm), aspirate 1 µL with a Hamilton syringe (Fig. 2E), and return remainder to ice.

#### Intracranial cell suspension injection

Lower the Hamilton microsyringe to the target coordinates (*X* = 2.3 mm, Y=midpoint between bregma and lambda, *Z* = 3.0 mm). Condition the injection tract by moving the syringe ±0.05 mm vertically 3-5 times. Reposition precisely at 3.0 mm depth. Initiate slow injection (0.5 µL/min) over 2 min. Upon completion of injection, maintain a 1-minute dwell period to minimize reflux along the needle track (reducing ectopic tumor formation risk).

#### Withdraw the Hamilton microliter syringe

Repeat step 2.14.

#### Closure of the skull and scalp

Seal the skull with bone wax and close the scalp with 3–4 simple interrupted sutures using a sterile, threaded needle.

#### Prevention of infection

After suturing is completed, disinfect the scalp again with povidone-iodine. Once dry, apply erythromycin ointment to the scalp.

#### Postoperative thermoregulation and recovery

House the mice in cages with thermostatic pads to maintain body temperature during anesthetic recovery.

### Nursing and follow-up after operation

#### Wound care

Observe the mouse’s wound daily and apply erythromycin ointment to the wound.

#### Postoperative monitoring and documentation

Mice should be monitored daily for the first three days post-operation for feeding, drinking, behavior, coat color, excretion, locomotor activity and posture, with any abnormalities thoroughly documented. Monitoring frequency can then be adjusted as needed.

#### Mouse weight recording

Record mouse weights daily for the first three days post-surgery; the interval may be extended thereafter as needed.

#### Mouse cranial condition record

Monitor the mouse’s head for bulging and record the time of onset.

#### Conditions and methods for euthanizing mice

For longitudinal observation, scheduled euthanasia will commence the second week post-implantation, with timing tailored to individual condition. Unscheduled euthanasia criteria encompass: severe cachexia (>25% weight loss within 48 h); neurological or motor impairments due to tumor mass effects (*e.g.*, kyphosis, paralysis, head tilt, inability to eat or drink normally, respiratory distress); and signs of dehydration such as poor skin turgor and sunken eyes. The procedure involves deep anesthesia with 1% pentobarbital sodium (50 mg/kg, i.p.), followed by cardiac perfusion, cervical dislocation, and brain collection.

### Verification methods for intracranial tumors

#### Small animal *in vivo* imaging

**Figure 5:**
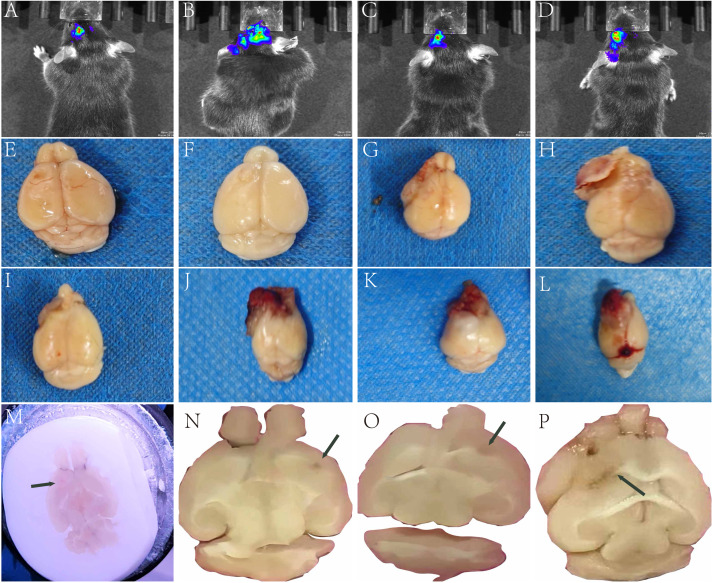
Gross images of intracranial tumor formation post-injection. (A–D) Intracranial tumorigenesis was induced by orthotopic injection of luciferase-overexpressing LLC cells in five mice, demonstrating an 80% survival rate (4/5) with 100% tumor formation efficiency as confirmed by bioluminescence imaging. (E–L) Orthotopic brain tumor models were established by intracranial implantation of LLC cells (*n* = 10). The cohort exhibited 100% survival and 100% tumor formation rates. Representative tumor progression images were captured at different time points: Groups (E–F) at 14 days post-implantation (dpi), (G–H) at 21 dpi, (I–K) at 25 dpi, and Group L at 29 dpi. M: Representative cryosection images obtained at 14 days post-implantation (14 dpi). (N–O) Representative paraffin-embedded tissue sections at 14 days post-implantation (14 dpi). (P) Representative paraffin-embedded tissue sections at 21 days post-implantation (21 dpi). The black arrow indicates the tumor.

By using luciferase-expressing tumor cell lines, tumors can be non-invasively monitored *via* BLI. Specifically, intraperitoneal injection of D-luciferin substrate allows for tumor localization using an *in vivo* imaging system after a formation period (Figs. 5A–5D).

#### Macroscopic examination of tumor-bearing brain tissue

Brain tissues collected at successive time points evealed tumor progression. At two weeks post-implantation, tumors were localized with relatively intact brain architecture (Figs. 5E–5F). By three weeks, masses protruded extracranially, invading the skull and often fusing with it (Figs. 5G–5H). Extended post-implantation periods resulted in significant necrosis and hemorrhage (Figs. 5I–5L).

#### Histological examination of mouse brain tissue

Prepare brain tissue cryosections (Fig. 5M) or paraffin-embedded sections (Figs. 5N–5P) for macroscopic examination, measurement, and recording of tumor location.

#### Microscopic observation of brain tissue sections

**Figure 6:**
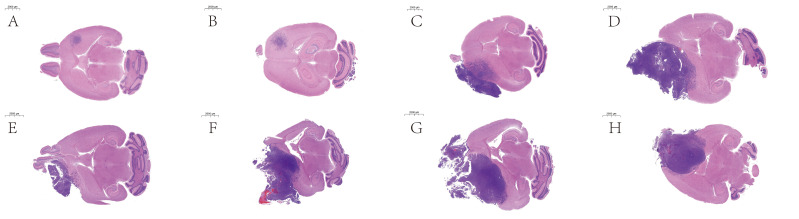
Histopathological sections of intracranial tumor models. Intracranial xenograft models were established using LLC cells. For longitudinal pathological evaluation, cohorts were randomly sacrificed at predetermined time intervals (14, 21, 25, and 29 days post-implantation). Following standard protocols, harvested brain tissues were fixed in 10% neutral buffered formalin, processed for paraffin embedding, and sectioned for hematoxylin and eosin (H&E) staining. The experimental groups were allocated as follows: (A–B) (14 dpi), (C–D) (21 dpi), (E–G) (25 dpi), and (H) (29 dpi).

Observe the anatomical position of tumor masses within murine brain tissue under the microscope after HE staining (Fig. 6).

## Results and Discussion

Intracranial injection technology enables precise implantation of tumor cells into specific brain regions of mice, establishing animal models that closely mimic the human brain tumor microenvironment, which holds significant importance for brain tumor research. However, existing literature reports varying injection sites, and there remains a lack of standardized protocols for reproducible intracranial tumor model establishment. This article summarizes a refined intracranial injection procedure (Fig. 3), providing in-depth exploration of key aspects including murine intracranial injection site selection and determination.

Previous studies have reported inconsistent intracranial tumor injection coordinates (Table 1), with insufficient methodological exploration and validation of site selection, casting doubt on the reproducibility of reported injection sites. To identify a suitable injection location for tumor induction in the striatum of C57BL mice, we first performed dye mapping experiments in 10 mice. This approach enabled immediate post-injection verification of targeting accuracy bypassing the 2–3 week waiting period required for tumor formation and dramatically reducing experimental duration. In the initial experiments, we injected dye at coordinates 2.3 mm left and one mm posterior to the bregma as described in previous literature (Li et al., 2023), but found all injection sites were positioned too anteriorly (Figs. 4A1–4C3). We then attempted injections two mm left and three mm deep to the lambda in two mice. Frozen section analysis revealed these injections were approximately one mm too posterior and had penetrated the ventricles (Figs. 4D1–4E3). To correct this, we adjusted the coordinates by moving one mm anteriorly from the lambda (two mm left, one mm anterior, and three mm deep to lambda), which successfully placed the injection sites precisely at the edge of the striatum (Figs. 4F1–4F3). However, using either bregma or lambda alone as reference points failed to account for individual variations in mouse anatomy due to factors like body weight. To minimize positional errors caused by individual differences, we developed a dual-reference system using both bregma and lambda as anatomical landmarks. Injections were performed 2.3 mm left or right and three mm deep relative to the midpoint between bregma and lambda. This approach was tested in four randomly selected mice of varying weights using dye injections. All injection sites were successfully positioned at the striatal edge, maintaining appropriate distances from both the ventricles and brain surface, thereby providing sufficient space for tumor growth (Figs. 4G1–4J3).

**Table 1:** Previously reported injection sites in the literature.

Literature	Mouse strain and size	Target sites and injection coordinates
Combination Radiotherapy in an Orthotopic Mouse Brain Tumor Model (2012), DOI: 10.3791/3397	The specific type and size of mice were not reported (images indicate nude mice)	The target site was not specified. The injection coordinates were 2 mm lateral (right), 1 mm anterior to bregma, with a depth of 3 mm from the skull surface.
Creating anatomically accurate and reproducible intracranial xenografts of human brain tumors (2014), DOI: 10.3791/52017	Male and female athymic nude mice aged 6–12 weeks (20 to 30 g)	The target site was the midbrain, with injection coordinates at 2.5 mm lateral (right), 1.5 mm anterior to bregma, and 3.5 mm ventral.
A method to accurately inject tumor cells into the caudate/putamen nuclei of the mouse brain (2004), PMID: 15717488	Female Balb/c nu/nu mice aged 4–6 weeks	The target site was the caudate/putamen, with injection coordinates at 0.5 mm anterior and 2 mm lateral to bregma, at a depth of 3.3 mm from the skull surface.
Establishment and Validation of CyberKnife Irradiation in a Syngeneic Glioblastoma Mouse Model (2021), DOI: 10.3390/cancers13143416	Female C57BL/6N mice aged 13.5 ± 3.5 weeks with an average body weight of 22.0 ± 1.2 g	The target site was the right striatum, with injection coordinates at 2 mm lateral and 1 mm anterior to bregma, at a depth of 3 mm.
Probiotics Alleviate Chemotherapy-Associated Intestinal Mucosal Injury via the TLR4–NFκB Signaling Pathway (2023), DOI: 10.2147/DDDT.S403087	8-10 week-old male C57BL/6 mice	The target site is the left putamen, with the injection coordinates set at 1 mm posterior to the bregma, 2.3 mm lateral, and 2.3 mm deep from the brain surface.
Quality Assessment of Stereotactic Radiosurgery of a Melanoma Brain Metastases Model Using a Mouselike Phantom and the Small Animal Radiation Research Platform (2017), DOI: 10.1016/j.ijrobp.2017.05.016	7-week-old male C57BL/6 mice	The target was not specified, with the injection site located at 1 mm anterior to the bregma, 2 mm lateral, and 2.5 mm deep from the brain surface.
Stereotactic injection of murine brain tumor cells for neuro-oncology studies (2025), DOI: 10.1016/bs.mcb.2024.07.005	No size reported, male or female C57BL/6 mice	The target site was the striatum, with the injection coordinates at 0.5 mm anterior to the bregma, 2 mm lateral to the sagittal suture, and 3.5 mm ventral to the dura mater.

**Figure 7:**
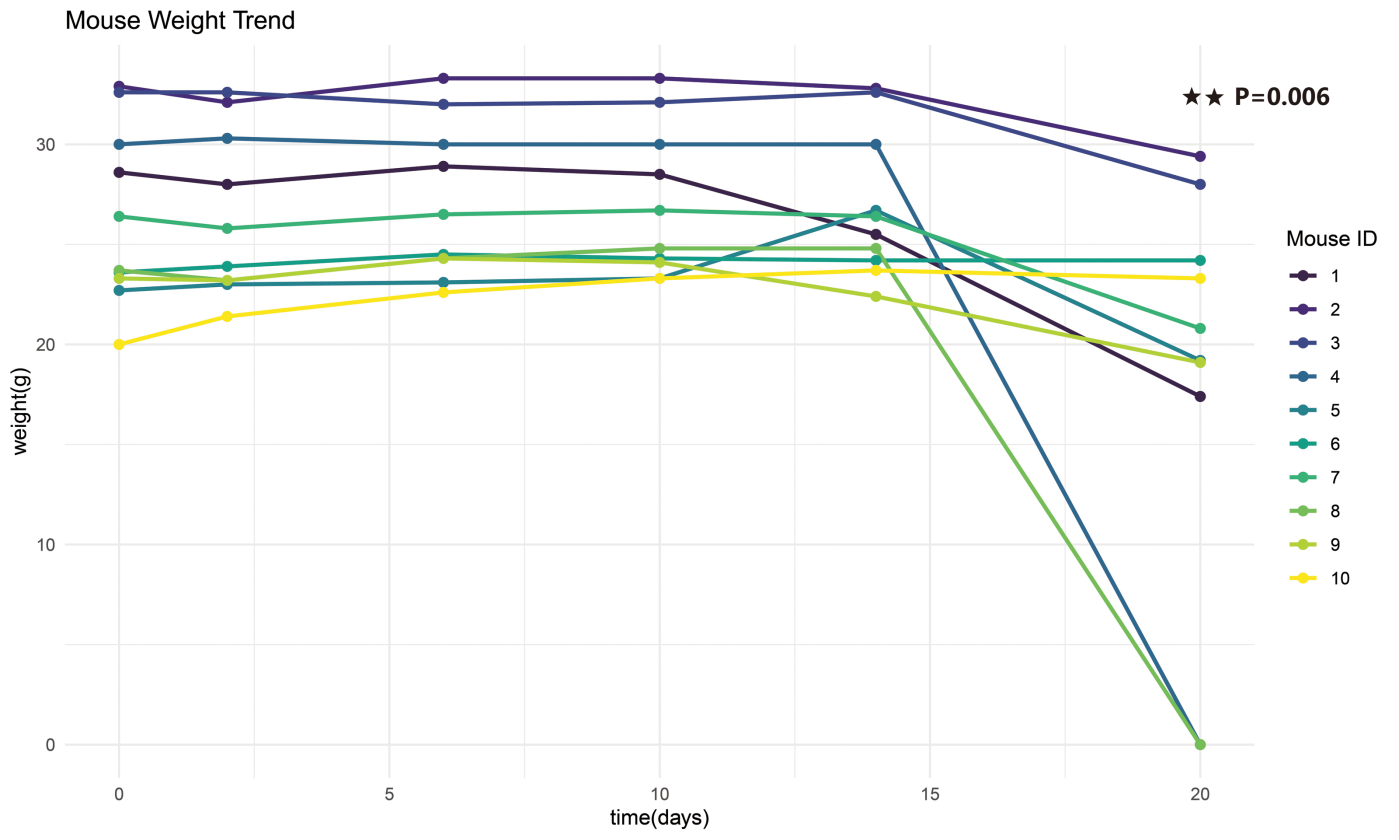
Temporal body weight fluctuation curve post-intracranial injection. Following intracranial inoculation of 2×10^5^ LLC cells in 10 C57BL/6 mice to establish orthotopic tumor models, body weight was monitored longitudinally. On day 15 post-implantation, two randomly selected mice were sacrificed after weight measurement. One-way ANOVA of all 10 mice showed no significant differences in body weight during the initial observation periods (day 0, 5, 10, and 15; SPSS25.0; *p* > 0.05). Paired *t*-test analysis of the remaining eight mice demonstrated significant weight reduction between day 14 and 20 (SPSS25.0; *p* = 0.006, Cohen’d=1.626).

Following preliminary dye injection tests to confirm optimal coordinates, we conducted tumorigenesis experiments by intracranially injecting 2 × 10^5^ LLC cells into 10 C57BL mice to evaluate: (1) survival rate, (2) tumor formation rate, (3) injection accuracy, and (4) preliminary tumor growth kinetics. Two mice were randomly sacrificed at 2 weeks, and their gross images (Figs. 5E–5F), paraffin sections (Figs. 5N–5O), and HE staining (Figs. 6A–6B) all confirmed the accuracy and reliability of tumor formation. In addition, another mouse frozen section (Fig. 5M) at 2 weeks also proved the accuracy of the method. The optimal timing for interventions such as radiotherapy and pharmacotherapy is within two weeks post-intracranial injection, as by the third week, the tumor volume rapidly increases, protruding outside the skull (Figs. 5G–5L; Figs. 6C–6H; Fig. S1C), and there is a general decline in body weight (Fig. 7). Given the relatively short expected lifespan of the control group at this stage (Fig. S2), interventions during this period may not yield effective comparative results.

During intracranial tumor inoculation, as time progresses, we may observe tumor protrusion from the brain surface over time (Fig. S1C). While this phenomenon is inevitable due to tumor’s natural tendency to expand toward areas of lower physical resistance (especially through the injection-created cranial defect), we aim to maintain the majority of tumor volume within the intracranial cavity through procedural refinements. By adjusting the procedural details, we can delay the onset of this protrusion. For instance, after the Hamilton syringe needle reaches the target depth of three mm, it can be further advanced 0.5 mm downward and held for several minutes before withdrawing to the three mm depth for injection, thereby allowing the tumor sufficient space to grow. Additionally, reducing the volume of the injected fluid and administering it at a slow, steady rate can help minimize backflow along the needle shaft. Studies by Yamada et al. have shown that the optimal tumor growth results from the smallest injection volume and rate, while Brooks et al. found that utilizing a microprocessor-controlled pump instead of manual injection offers greater reproducibility and efficiency (Brooks et al., 1998; Yamada et al., 2004). However, a slower injection rate may prolong the procedure, which is less favorable for large-scale animal modeling studies that require efficiency and speed.

During the dye injection validation process, we used two types of dyes: Coomassie Brilliant Blue (Figs. 4A1–4I3) and Methylene Blue (Figs. 4J1–4J3). The study showed that Coomassie Brilliant Blue has minimal peripheral penetration and higher injection accuracy, which aids in precisely determining the injection site. In contrast, Methylene Blue exhibits stronger tissue penetration, with the dye spreading across the injection site. Previous studies (Yamada et al., 2004) that utilized Indigo Carmine to investigate intracranial injection rates and volumes revealed, upon image comparison, that this dye has significantly higher tissue permeability than Coomassie Brilliant Blue. Therefore, Coomassie Brilliant Blue can serve as an excellent dye for localization studies in intracranial injections.

During mouse anesthesia, we used 1% sodium pentobarbital for intraperitoneal injection at a dose of 50 mg/kg. However, this anesthetic protocol exhibited prolonged induction time, shallow anesthesia depth, and inconsistent efficacy with some mice showing inadequate anesthetic effects. Additionally, this concentration makes dosage control difficult, increasing the risk of severe adverse reactions such as respiratory depression and bradycardia caused by overdose, even leading to mouse death. To prevent such incidents, the dosage and incremental rules of the anesthetic should be strictly controlled based on the mouse’s body weight. Furthermore, reducing the anesthetic concentration could provide greater flexibility in adjusting the dosage. In other researchers’ intracranial injection protocols, various anesthesia methods have been employed, including isoflurane inhalation (Pierce & Keating, 2014), intraperitoneal injection of chloral hydrate (Li et al., 2023), and a combination of ketamine and xylazine for intraperitoneal injection (Hunn et al., 2012; Jelgersma et al., 2021). The combination of ketamine hydrochloride and xylazine can rapidly induce anesthesia, allowing mice to enter an anesthetized state quickly while providing effective analgesia and a wider safety margin for anesthesia. Inhalation anesthesia, on the other hand, enables rapid induction, allowing mice to reach the desired anesthesia level in a short time with easily adjustable depth, thereby reducing anesthesia-related complications. When experimental conditions permit, these alternative anesthesia methods may also be adopted for the study.

**Figure 8:**
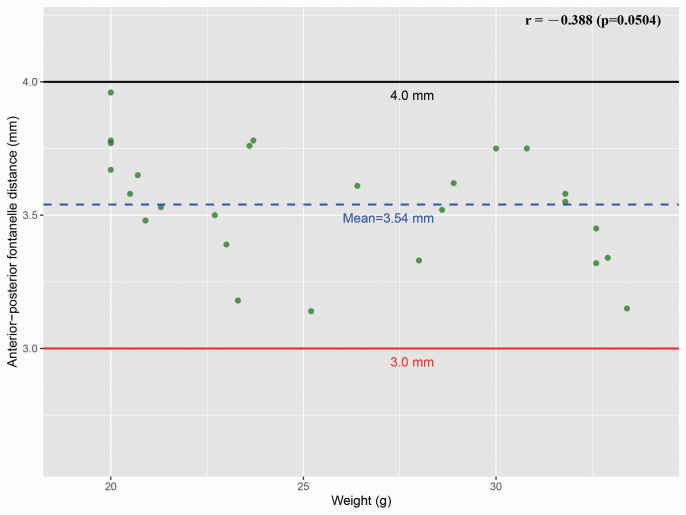
Correlation between body weight and bregma-lambda distance. A scatter plot illustrating the relationship between body weight and the anterior-posterior fontanelle distance in 26 mice showed that all measurements of fontanelle distance were greater than 3.0 mm and less than 4.0 mm, with an average of 3.54 mm. The Pearson correlation coefficient was −0.388, and the *p*-value was greater than 0.05, indicating that there is no statistically significant linear correlation between the two variables (SPSS25.0). As shown in the figure, body weight and anterior-posterior fontanelle distance do not exhibit a linear increasing relationship. Mice with smaller body weights did not show significant differences in fontanelle distance compared to those with larger weights; both groups fluctuated around an average of approximately 3.54 mm.

This study found that the posterior fontanelle was difficult to identify in a small subset of mice, whereas the anterior fontanelle was relatively easy to locate (Figs. S1E–S1H). We also recorded the body weight and the anterior-posterior fontanelle distance (AFD) in these mice. Interestingly, the AFD in C57BL/6 mice did not increase linearly with body weight. Instead, among 26 mice (20 g–33.4 g), the mean AFD was 3.54 mm. Regardless of body weight, the AFD in all mice fluctuated around 3.54 mm, with upper and lower limits of four mm and three mm, respectively. Half of the AFD (AFD/2) ranged between 1.5 mm and 2.0 mm, with a mean of 1.77 mm (Fig. 8). These findings suggest that the AFD in C57BL/6 mice falls within a precise and narrow range. This has significant implications for intracranial injections, particularly in cases where the posterior fontanelle is difficult to identify. Since the anterior fontanelle is easily recognizable, a point approximately 1.77 mm (1.5–2.0 mm) posterior to it can serve as a reliable approximation for midpoint of bregma and lambda, independent of the mouse’s body weight.

This study has several limitations. First, the research was conducted exclusively on female C57BL/6 mice and did not investigate male C57BL/6 mice and other mouse strains. Given the known anatomical differences in brain structure, whether this injection site selection method can be generalized to male C57bl/6 mice or other strains remains uncertain. However, this study provides a standardized approach that may serve as a reference for intracranial injection studies in other mouse varieties. Additionally, this study was limited to C57BL/6 mice weighing between 20–33.4 g. The relationship between anterior-posterior fontanelle distance (AFD) and body weight remains unclear for mice outside this weight range. It is possible that the standardized injection site (1.77 mm posterior to the anterior fontanelle) may not be directly applicable to significantly smaller (<20 g) or larger (>33.4 g) mice, as their cranial anatomy and AFD-body weight correlation could differ. Finally, due to differences in population genetic structure caused by genetic drift and natural selection (Lynch et al., 2016), the authors emphasize that even when using mice of the same strain for research, the standardized injection site exploration and validation procedures outlined in this study should be followed. One should not directly use injection sites validated by others without proper verification.

## Conclusion

In summary, we found the bregma-lambda distance in C57BL/6 mice fluctuates around 3.54 mm and non-linearly increases with body weight, and established a protocol for intracranial orthotopic tumor modeling.

## Supplemental Information

10.7717/peerj.20913/supp-1 Supplemental Information 1 Some details of the experiment A-B: The appropriate anatomical plane was selected, and the dye localization was measured using a calibrated ruler. C: The white arrows indicate the protruding mass on the mouse’s skull four weeks after intracranial injection of LLC cells. D-E: Bleeding during drilling. E-H: Images for distinguishing the anterior and posterior fontanelles. The white arrow indicates the anterior fontanelle, and the black arrow indicates the posterior fontanelle. E-F: Easier to identify the anterior fontanelle; G: Difficult to distinguish the posterior fontanelle; H: Identification of the posterior fontanelle was not feasible

10.7717/peerj.20913/supp-2 Supplemental Information 2 KM Survival Curves of Tumor-bearing and Control Groups This study pooled data from multiple recent experiments (excluding mice that did not meet the euthanasia criteria but were euthanized as scheduled) to generate survival curves for the tumor-bearing group (injected with a PBS suspension of LLC cells) and the control group (injected with PBS only). As shown in the figure, all mice in the tumor-bearing group survived for more than three weeks, after which the survival rate dropped sharply, with very few individuals surviving beyond 30 days. In contrast, all mice in the control group exhibited long-term survival.

10.7717/peerj.20913/supp-3 Supplemental Information 3 Cardiac perfusion and brain extraction;Brain Tissue Cryosectioning This document describes the procedure for obtaining brain tissue via mouse cardiac perfusion, as well as the steps for frozen sectioning of mouse brain tissue.

10.7717/peerj.20913/supp-4 Supplemental Information 4 Raw Data: Body weight and anterior-posterior fontanelle distance

10.7717/peerj.20913/supp-5 Supplemental Information 5 Raw Data-Weight Record Form

10.7717/peerj.20913/supp-6 Supplemental Information 6 ARRIVE 2.0 checklist

10.7717/peerj.20913/supp-7 Supplemental Information 7 Lentiviral Core Plasmid The sequence of the lentiviral core plasmid for luciferase overexpression.

10.7717/peerj.20913/supp-8 Supplemental Information 8 Quality inspection report and STR identification report for LLC cells

## Additional Information and Declarations

### Data Availability

The following information was supplied regarding data availability: The raw measurements are available in the Supplementary File.
