# Supplementary material for: Establishment and validation of an orthotopic brain metastasis tumor model in C57BL/6 mice
Source: PeerJ. 2026 Mar 26;14:e20913. doi: 10.7717/peerj.20913 (PMC13033286; doi:10.7717/peerj.20913)
Supplement: Supplemental Information 1 — A-B: The appropriate anatomical plane was selected, and the dye localization was measured using a calibrated ruler. C: The white arrows indicate the protruding mass on the mouse’s skull four weeks after intracranial injection of LLC cells. D-E: Bleeding during drilling. E-H: Images for distinguishing the anterior and posterior fontanelles. The white arrow indicates the anterior fontanelle, and the black arrow indicates the posterior fontanelle. E-F: Easier to identify the anterior fontanelle; G: Difficult to distinguish the posterior fontanelle; H: Identification of the posterior fontanelle was not feasible [file peerj-14-20913-s001.docx]

# Supplemental Fig S1

Supplementary Fig S1. Some details of the experiment


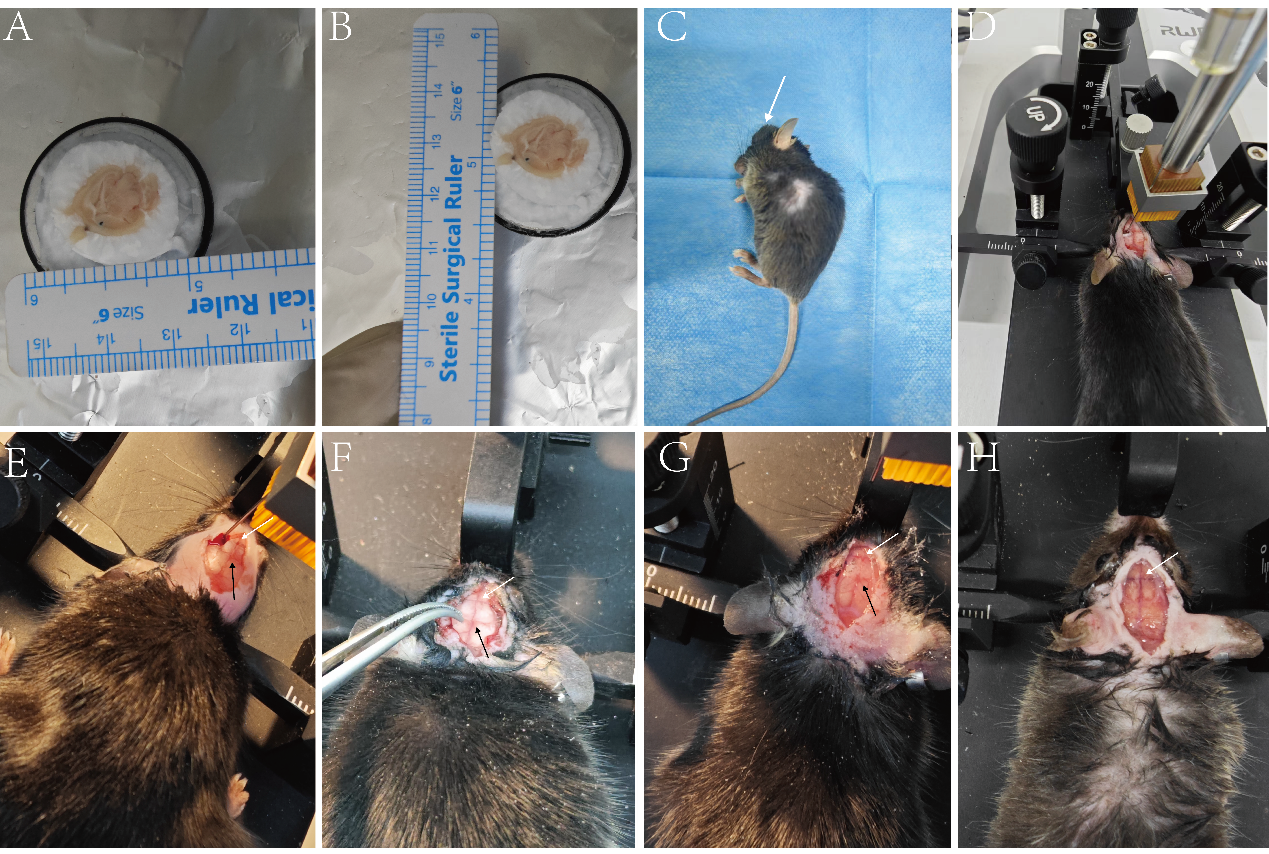


A-B: The appropriate anatomical plane was selected, and the dye localization was measured using a calibrated ruler. C: The white arrows indicate the protruding mass on the mouse's skull four weeks after intracranial injection of LLC cells. D-E: Bleeding during drilling. E-H: Images for distinguishing the anterior and posterior fontanelles. The white arrow indicates the anterior fontanelle, and the black arrow indicates the posterior fontanelle. E-F: Easier to identify the anterior fontanelle; G: Difficult to distinguish the posterior fontanelle; H: Identification of the posterior fontanelle was not feasible
