# Supplementary material for: Establishment and validation of an orthotopic brain metastasis tumor model in C57BL/6 mice
Source: PeerJ. 2026 Mar 26;14:e20913. doi: 10.7717/peerj.20913 (PMC13033286; doi:10.7717/peerj.20913)
Supplement: Supplemental Information 2 — This study pooled data from multiple recent experiments (excluding mice that did not meet the euthanasia criteria but were euthanized as scheduled) to generate survival curves for the tumor-bearing group (injected with a PBS suspension of LLC cells) and the control group (injected with PBS only). As shown in the figure, all mice in the tumor-bearing group survived for more than three weeks, after which the survival rate dropped sharply, with very few individuals surviving beyond 30 days. In contrast, all mice in the control group exhibited long-term survival. [file peerj-14-20913-s002.docx]

# Supplemental Fig S2

Supplementary Fig S2. KM Survival Curves of Tumor-bearing and Control Groups


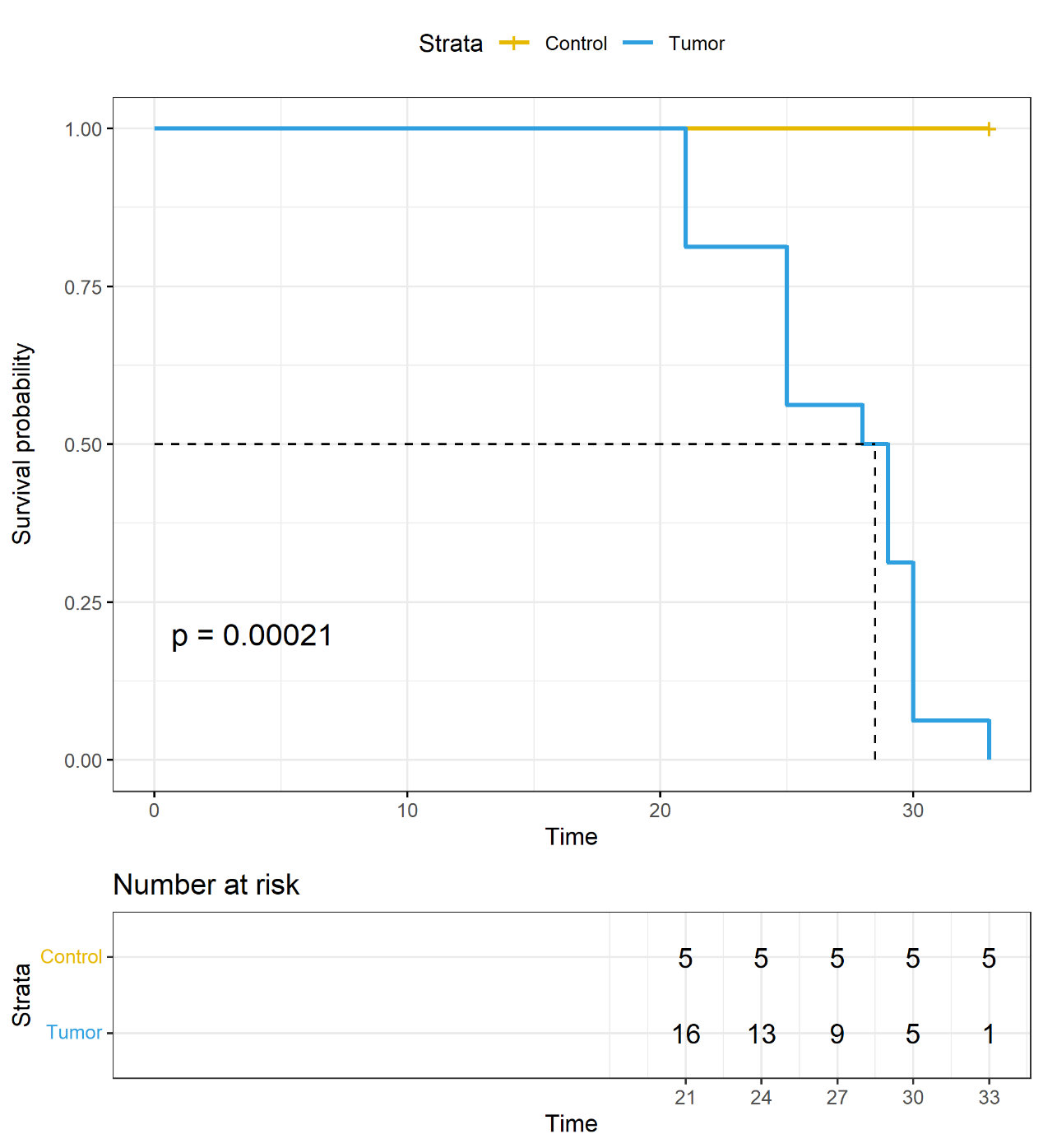


Note: This study pooled data from multiple recent experiments (excluding mice that did not meet the euthanasia criteria but were euthanized as scheduled) to generate survival curves for the tumor-bearing group (injected with a PBS suspension of LLC cells) and the control group (injected with PBS only). As shown in the figure, all mice in the tumor-bearing group survived for more than three weeks, after which the survival rate dropped sharply, with very few individuals surviving beyond 30 days. In contrast, all mice in the control group exhibited long-term survival.
