# Supplementary material for: Establishment and validation of an orthotopic brain metastasis tumor model in C57BL/6 mice
Source: PeerJ. 2026 Mar 26;14:e20913. doi: 10.7717/peerj.20913 (PMC13033286; doi:10.7717/peerj.20913)
Supplement: Supplemental Information 3 — This document describes the procedure for obtaining brain tissue via mouse cardiac perfusion, as well as the steps for frozen sectioning of mouse brain tissue. [file peerj-14-20913-s003.pdf]

Supplemental Article S1

Cardiac perfusion and brain extraction

Brain Tissue Cryosectioning

Cardiac perfusion and brain extraction

1.Mouse anesthesia (if anesthesia is good, can enter the next step)： Administer 1% sodium pentobarbital intraperitoneally (i.p.) at 0.1 mL/20 g body weight (50 mg/kg). Assess anesthesia efficacy 5–10 minutes post-injection by pinching the hind limb with hemostatic forceps. If the anesthetic effect is insufficient, administer an additional dose of 0.03mL/20 g (equivalent to 15mg/kg), reassess after 5 minutes, and repeat the process as needed.

2.Liquid preparation: 20ml perfusion salt solution and 20ml paraformaldehyde were extracted using a 20ml syringe.

3. Exposure of Mouse Heart and Liver: The abdomen of the mouse was upward. Make an inverted V-shaped incision through the skin to expose the abdomen, diaphragm, and thoracic cage. Gently separate the diaphragm from the thoracic wall to avoid cardiac injury. Cut the ribs bilaterally and reflect them upward. Use ophthalmic forceps to carefully dissect the pericardial fat, fully exposing the heart.

4.Create the perfusion channel : Insert a disposable intravenous infusion needle approximately 3–4 mm into the left ventricular apex and secure it (avoid penetrating the left ventricle completely). Using ophthalmic scissors, make an incision at the darkened region superior to the right ventricle (right atrial appendage) to allow drainage of dark red venous return blood (Supplementary Fig. 1I–J).

5. Anticoagulant perfusion with saline solution: The assistant connects the perfusion saline syringe to the infusion needle and injects slowly. During this process, the liver color will transition to a pale hue and effluent from the right auricle will progressively lighten. After the outflow was completely clarified, the heparinization was essentially completed(Supplementary Fig. 1J-K).

6. Paraformaldehyde Perfusion Fixation: Connect the paraformaldehyde syringe to the infusion needle and administer slowly. In this process, the limbs of the mice can be observed twitching and the tail swinging, which indicates that the perfusion channel is well constructed. Upon completion of perfusion, the mouse body becomes rigid. When lifting by the tail, the torso exhibits complete stiffness, indicating successful fixation (Supplementary Fig. 1L).

7. Brain tissue exposure: Sever the connection between the cervical vertebrae and skull, then remove the head. Incise the scalp to fully expose the skull. Using forceps, carefully peel the skull outward starting from the foramen magnum, taking care to avoid damaging brain tissue.

8. Brain tissue isolation: Gently lift the brain tissue upward from the cerebellum region, carefully sever the cranial nerves, and transfer the isolated brain into 4% paraformaldehyde solution for overnight fixation.

Brain Tissue Cryosectioning

1.Sucrose Immersion: Remove the brain tissue from the fixative and immerse it in 30% sucrose solution at 4°C for 2 hours.

2. OCT Embedding: Select an appropriately sized embedding mold or shape aluminum foil to create a suitable container. First, add a small amount of OCT embedding medium. After absorbing excess sucrose solution from the brain tissue surface, place the tissue horizontally in the mold. Then, add more OCT medium until the tissue is completely submerged.

3. Flash Freezing: Place the embedding mold on a pre-cooled freezing stage. After the OCT compound turns white, apply a small amount of OCT to the sample holder. Transfer the embedded sample from the mold to the holder and position it on the freezing stage.

4. Trimming Sections: Install the blade at a 5-7° angle and mount the sample holder onto the microtome head. Set the section thickness to approximately 50 μm and trim until reaching the desired tissue plane.

5. Slicing : Adjust the thickness of a single slice to about 5-15μm, put down the anti-roll plate, slice (when cut to the corresponding level, take a picture to record the position of the dye), lift the anti-roll plate, and stick the slice with a glass slide.
