# Supplementary material for: Establishment and validation of an orthotopic brain metastasis tumor model in C57BL/6 mice
Source: PeerJ. 2026 Mar 26;14:e20913. doi: 10.7717/peerj.20913 (PMC13033286; doi:10.7717/peerj.20913)

Quality inspection report and STR identification report for LLC cells. The quality inspection report includes mycoplasma testing results.

​**​LLC Quality Inspection Report​**​


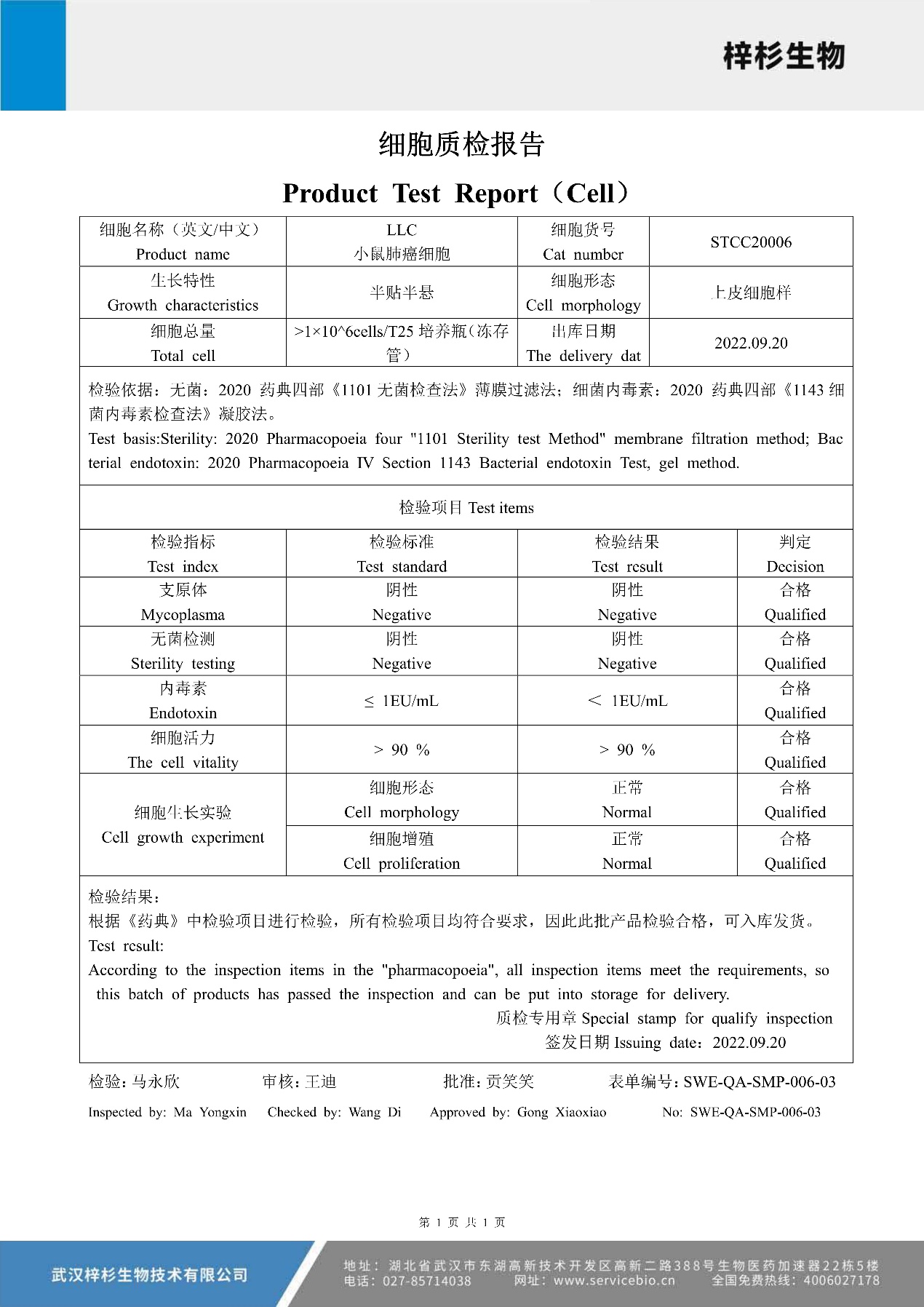


**LLC** **STR identification report**


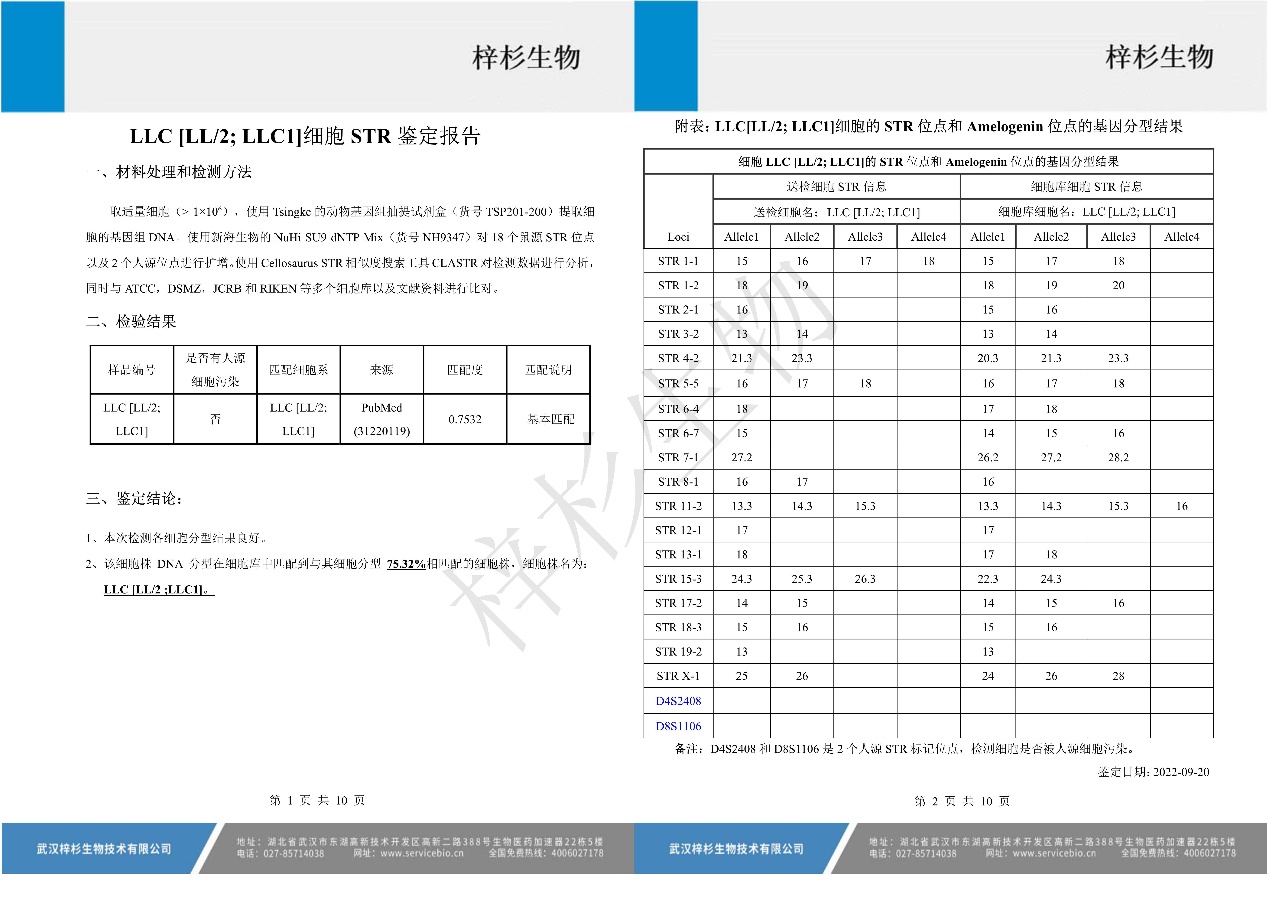

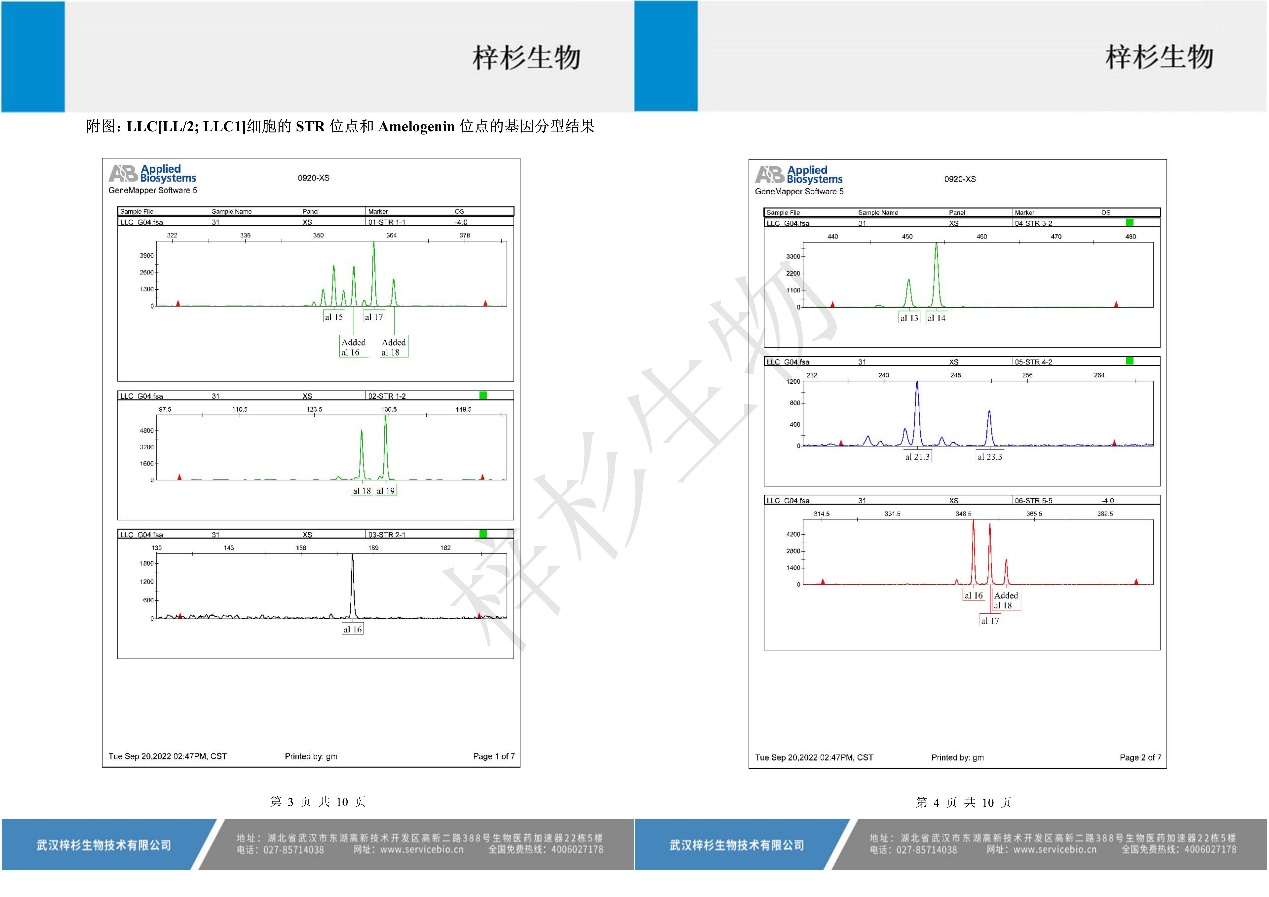

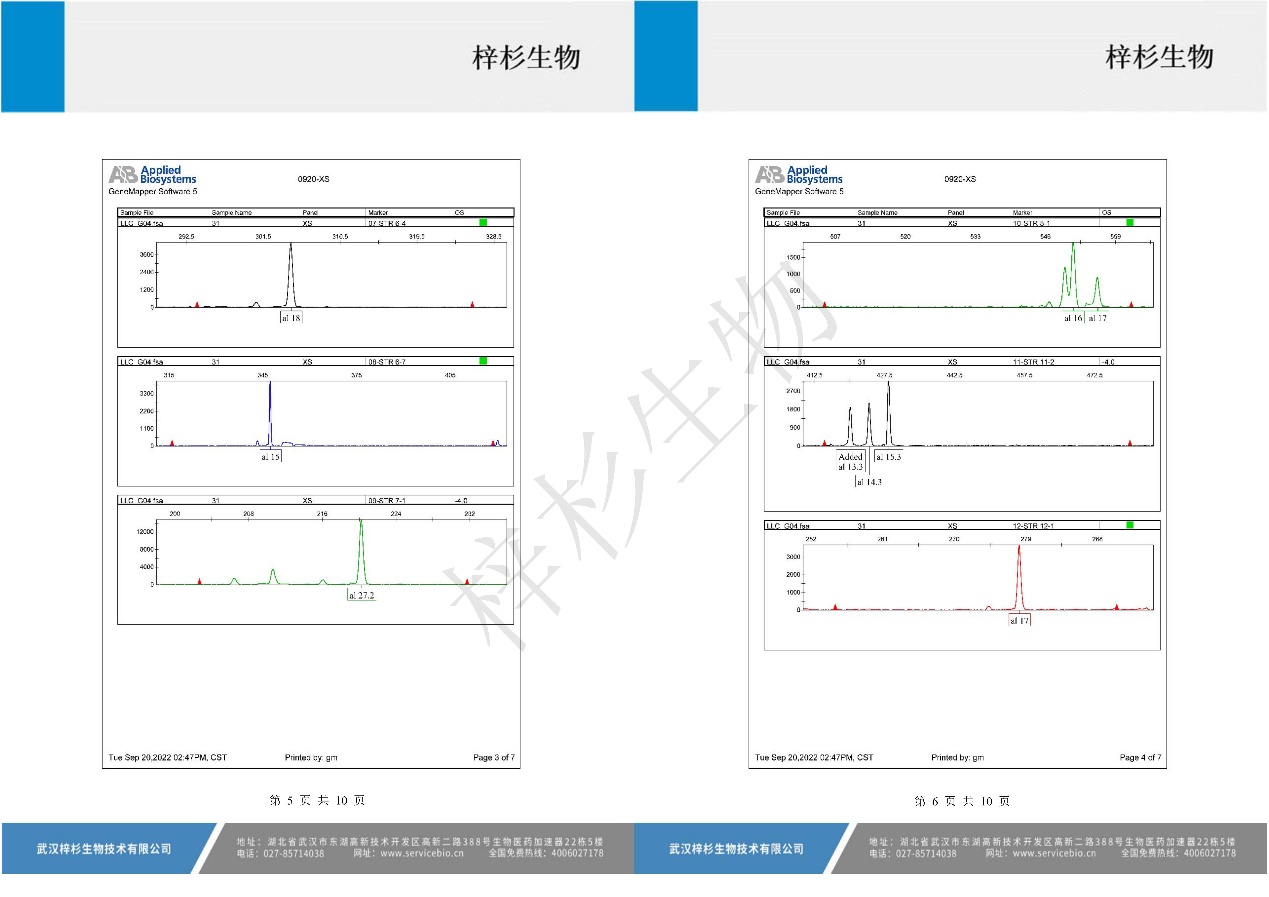

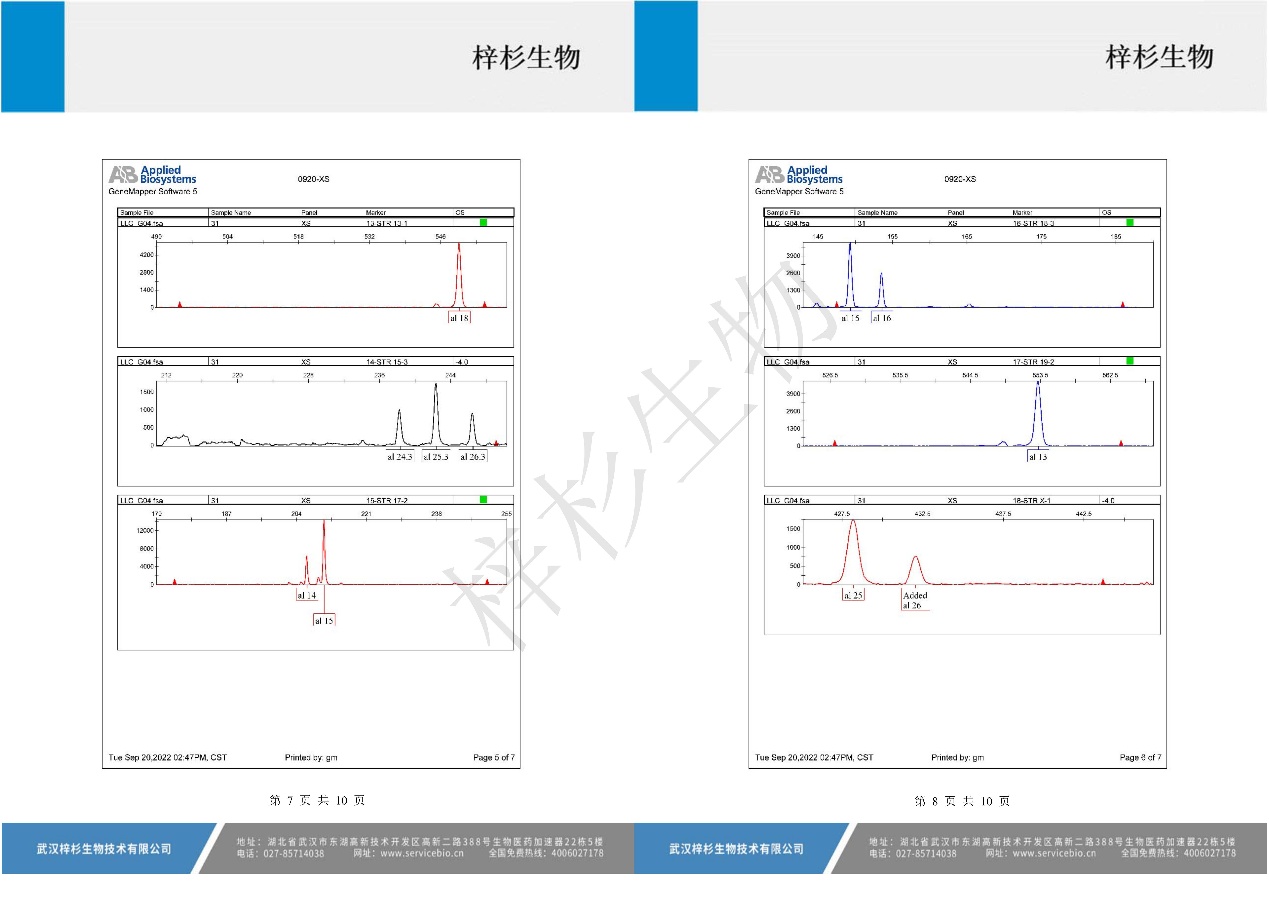

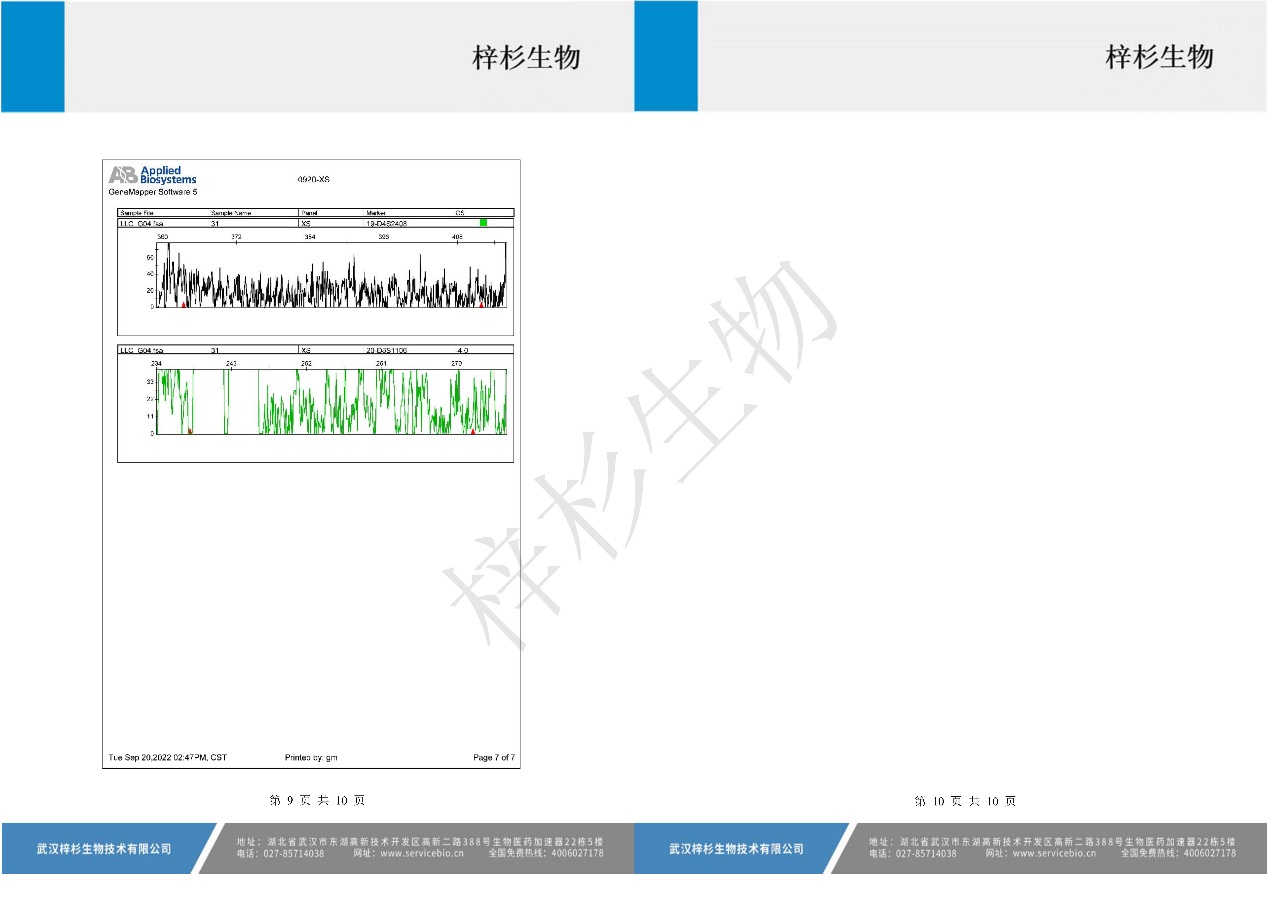

Supplement: Supplemental Information 8 [file peerj-14-20913-s008.docx]
